# Learning Curve of Perclose ProGlide Utilization During Percutaneous Coronary Intervention

**DOI:** 10.7759/cureus.38155

**Published:** 2023-04-26

**Authors:** Eser Varis, Dogac Caglar Gurbuz

**Affiliations:** 1 Department of Cardiology, Private Istanbul Hospital, Istanbul, TUR; 2 Department of Cardiology, Koc University, Istanbul, TUR

**Keywords:** success, perclose proglide™, learning curve, coronary intervention, complication

## Abstract

Objective: This study aimed to investigate the learning curve (LC) of Perclose ProGlide (Chicago, IL: Abbott Laboratories) utilization for percutaneous coronary intervention (PCI) for the first time.

Methods: The study was conducted in a prospective manner and the final sample of the study was determined as 80 patients. Patients’ characteristics, diameter of common femoral artery (CFA), distance from skin to CFA, degree of calcification (<50% or ≥50%), procedure-related parameters, complications, and success of procedures were recorded. Patients were equally divided into four groups and groups were compared according to patient demographic properties, procedure-related parameters, complications, and success.

Results: The mean age and mean BMI of the study population were 55.5 years and 27.5 kg/m^2^, respectively. The mean procedure time was 144.8 minutes (min) in group 1, 138.9 min in group 2, 122.2 min in group 3, and 101.1 min in group 4, and the difference was statistically shorter in favor of group 3 and group 4 (p=0.023). Moreover, mean fluoroscopy time significantly decreased after 20 cases (p=0.030). Hospitalization period was significantly shortened following 40 procedures (p=0.031). Complications were detected in five patients in group 1, four patients in group 2, and one patient in group 4 (p=0.044). Success was significantly higher in group 3 and group 4 in comparison to group 1 and group 2 (p=0.040).

Conclusion: This study showed that procedure time and hospitalization time significantly decreased after 40 cases and fluoroscopy time significantly decreased after 20 cases. Moreover, after 40 procedures, the success of Perclose ProGlide utilization during PCI significantly increased and complications of the procedure significantly decreased.

## Introduction

Percutaneous coronary intervention (PCI) is frequently performed minimally invasive procedure to evaluate coronary vessels and to treat stenosis of coronary arteries. Although PCI has a satisfying success rate and acceptable complication rate, previous studies emphasized that bleeding is associated with increases in morbidity, hospitalization time, healthcare costs, and mortality [1]. Ellis et al. analyzed data from patients who underwent PCI, and the authors found that death rate of 10.4 in patients with major bleeding. They concluded that bleeding increased the mortality rate by 14.8-fold [2]. In another study, Arora et al. demonstrated that mortality increased four times in patients with major bleeding compared to patients without major bleeding after PCI [3]. The Perclose ProGlide (Chicago, IL: Abbott Laboratories) is one of the preferred closure devices after PCI. Although the efficiency and safety of Perclose ProGlide were proven by numerous studies, the relative technical difficulty of the procedure limits its extensive use in some cardiology centers [4].

The learning curve (LC) is simply defined as the case number that is required for an operator to achieve sufficient success rate with tolerable complication rates for a specific procedure [5]. To organize resident training programs and to determine the number of procedures that should be done in clinic supervision programs, identifying LC is critical for each intervention.

Although numerous studies showed the efficiency and reliability of Perclose ProGlide in PCI, to the best of our knowledge, no study has analyzed the LC for Perclose ProGlide use. In the present study, for the first time, the LC of Perclose ProGlide utilization was investigated for PCI.

## Materials and methods

The study was conducted in a prospective manner and the final sample of the study was determined as 80 patients. Power analysis was performed by evaluating the data of the reference literature on the subject, it was calculated that a total of 80 patients should be included, with a 95% confidence interval and a 20% margin of error [6]. The study started in December 2021 and finished in December 2022. Informed, written consent was obtained from all patients with detailed discussions pertaining to procedure details. Patients who underwent coronary angiography with Perclose ProGlide were enrolled in the study. All cases were performed by a single cardiologist, who had 10 years of experience working in the minimal invasive cardiology laboratory and >5000 coronary angiography cases. Moreover, the cardiologist was certified for Peeclose after attendance of a two-day dry laboratory and experimental course. The indication for Perclose ProGlide was access to the common femoral artery in cases who underwent PCI. If the puncture site was located above the inguinal ligament or above the inferior border of the inferior epigastric artery, Perclose ProGlide was not used due to possible risk of retroperitoneal hematoma, so these patients were excluded from the study. Also, patients with multiple access in the same location, patients with puncture to superficial femoral artery or the profunda femoris artery, and patients with puncture through the posterior wall were excluded. Lastly, morbidly obese patients, patients who underwent pelvic radiation therapy, and patients under 18 years of age were not enrolled in the study.

Age, sex, body mass index (BMI), presence of hypertension, and diabetes mellitus were noted for each patient. In addition, diameter of common femoral artery (CFA), distance from skin to CFA, degree of calcification (<50% or ≥50%), complications, and success of procedures were recorded. In addition, procedure-related parameters including procedure time, fluoroscopy time, and hospitalization period were noted. Complications were categorized as minor and major.

All procedures were performed in the same manner as previously described [7]. Cases that could not achieve vessel closure with Perclose ProGlide and cases requiring another vessel closure system in addition to Perclose ProGlide were accepted as unsuccessful procedures. A total of 80 patients were enrolled in the study, and patients were equally divided into four groups (the first 20 Perclose ProGlide cases were classified as group 1, and last 20 Perclose ProGlide cases were enrolled in group 4). The four groups were compared according to patient demographic properties, procedure-related parameters, complications, and success.

Statistical analysis

The SPSS version 26 (Armonk, NY: IBM Corp.) program was utilized during statistical analysis. The Shapiro-Wilk test was performed to evaluate normality of variable distribution. The ANOVA test was used for comparison of continuous variables between the four groups. For post-hoc analysis, the Tukey test was used, and for categorical parameters, Fisher’s exact test was used. Quantitative data are described as mean±standard error values. The data were analyzed at 95% confidence level and p-value of less than 0.05 was considered statistically significant.

## Results

In total, 80 patients (20 patients for each group) were enrolled in the study. The mean age and mean BMI of the study population were 55.5 years and 27.5 kg/m^2^, respectively. Of all participants, 65.0% (52 patients) were male. The mean CFA diameter was 8.1 mm for participants. The mean distance from skin to CFA was 30.1 mm, and 31 (29.7%) patients had a calcification degree ≥50%. Bleeding occurred in 10 (12.5%) patients, and success of procedure was 96.3% (77 patients) in the study (Table 1).

**Table 1:** Demographic data for study participants. *Mean±standard deviation. ISTH: International Society on Thrombosis and Haemostasis bleeding scale; DM: diabetes mellitus; CFA: common femoral artery

Variables		N=80
Age (years)*		55.5±8.9
Sex, n (%)	Male	52 (65.0%)
	Female	28 (35.0%)
BMI (kg/m²)*		27.5±5.1
Comorbidities, n (%)	Hypertension	61 (76.3%)
	DM	27 (33.8%)
	CFA diameter (mm)*	8.1±1.1
	Distance from skin to CFA (mm)*	30.1±5.9
Degree of calcification, n (%)	<50%	49 (61.3%)
	≥50%	31 (29.7%)
	Success, n (%)	77 (96.3%)
	Bleeding (ISTH), n (%)	10 (12.5%)

Comparison of the four groups with regards to patient characteristics revealed that age, sex, BMI, presence of hypertension, and diabetes mellitus were similar between the four groups (p=0.439, p=0.932, p=0.755, p=0.859, and p=0.680, respectively). In addition, CFA diameter, distance from skin to CFA, and degree of calcification were comparable between the four groups (p=0.484, p=0.463, and p=0.901). Comparison of the four groups with regard to pre-procedural properties is presented in Table 2.

**Table 2:** Comparison of pre-operative demographic data between groups. *Mean±standard deviation. DM: diabetes mellitus; CFA: common femoral artery

Variables		Group 1 (n=20)	Group 2 (n=20)	Group 3 (n=20)	Group 4 (n=20)	p-Value
Age (years)*		54.9±10.4	59.2±7.3	56.1±9.2	55.6±8.4	0.439
Sex, n (%)	Male	12 (60.0%)	13 (65.0%)	14 (70.0%)	13 (65.0%)	0.932
	Female	8 (40.0%)	7 (35.0%)	6 (30.0%)	7 (35.0%)	
BMI (kg/m²)*		27.2±4.7	26.9±5.5	27.5±5.0	28.6±5.6	0.755
Comorbidities, n (%)	Hypertension	15 (75.0%)	14 (70.0%)	16 (80.0%)	16 (80.0%)	0.859
	DM	6 (30.0%)	8 (40.0%)	5 (25.0%)	8 (40.0%)	0.680
CFA diameter (mm)*		8.2±1.2	7.9±1.1	8.4±0.9	8.1±1.2	0.484
Distance from skin to CFA (mm)*		29.6±6.4	30.8±5.3	31.5±5.7	28.7±6.3	0.463
Degree of calcification, n (%)	<50%	11 (55.0%)	13 (65.0%)	13 (65.0%)	12 (60.0%)	0.901
	≥50%	9 (45.0%)	7 (35.0%)	7 (35.0%)	8 (40.0%)	

The mean procedure time was 144.8 minutes (min) in group 1, 138.9 min in group 2, 122.2 min in group 3, and 101.1 min in group 4, and the difference was statistically shorter in favor of group 3 and group 4 (p=0.023). Moreover, mean fluoroscopy time significantly decreased after 20 cases (213.2 s for group 1, 150.8 s for group 2, 142.8 s for group 3, and 122.2 s for group 4, p=0.030). Also, hospitalization period was significantly shortened following 40 procedures (p=0.031). The post-procedure bleeding status of the patients was evaluated using the International Society on Thrombosis and Hemostasis bleeding scale (ISTH). Minor bleeding was observed in four patients in group 1 and in two patients in group 2. The count of major bleeding was one patient in group 1, two patients in group 2, and one patient in group 4 (p=0.044). Success was significantly higher in group 3 and group 4 in comparison with group 1 and group 2 (p=0.040) (Table 3).

**Table 3:** Comparison of operative parameters and post-operative results between groups. ^*^Mean±standard deviation. ^**,***^Statistically significantly different results between paired groups. ISTH: International Society on Thrombosis and Hemostasis

Variables		Group 1 (n=20)	Group 2 (n=20)	Group 3 (n=20)	Group 4 (n=20)	p-Value
Procedure time (minutes)^*^		144.8±47.8^**^	138.9±50.1^**^	122.2±59.0^***^	101.1±30.9^***^	0.023
Fluoroscopy time (seconds)^*^		213.2±95.4^**^	150.8±107.9^***^	142.8±99.8^***^	122.2±94.0^***^	0.030
Hospitalization time (hours)^*^		101.9±36.2^**^	102.1±34.7^**^	85.1±34.7^***^	72.1±41.2^***^	0.031
Success, n (%)	Success	18 (90.0%)^**^	16 (90.0%)^**^	19 (95.0%)^***^	20 (100.0%)^***^	0.040
	Failure	2 (10.0%)	2 (10.0%)	1 (5.0%)	-	
Bleeding (ISTH), n (%)		5 (25.0%)^**^	4 (20.0%)^**^	-^***^	1 (5.0%)^***^	0.044
Minor		4 (20.0%)	2 (10.0%)	-	-	
Major		1 (5.0%)	2 (10.0%)	-	1 (5.0%)	

## Discussion

The definition of LC is unique for each procedure, and defining LC is useful to determine procedure numbers and training program to achieve surgical proficiency. Also, defining LC can be used to gain the right to do the procedure. Authors have previously investigated the LC of different procedures; however, to our knowledge, no study analyzed the LC for Perclose ProGlide utilization during PCI [8,9]. In the present study, for the first time, it was demonstrated that procedure time and hospitalization time significantly decreased after 40 procedures, and fluoroscopy time significantly decreased following 20 procedures. In addition, after 40 cases, the success of Perclose ProGlide utilization during PCI significantly increased and complications of the procedure significantly decreased.

The aim of Perclose ProGlide is to achieve adequate vessel closure and bleeding control [10]. The role of LC in medical procedure success was evaluated in different health disciplines. Eesa et al. analyzed the correlation between LC and angiographic recanalization success of brain vessels in stroke and achieved a plateau in success after 50 procedures [11]. In a different study, Baytaroglu and Sevgili investigated the LC for percutaneous thrombectomy in the management of deep vein thrombosis of the lower limb, and the authors concluded that success significantly increased after 20 procedures [12]. In this study, for the first time, we found that the success of Perclose ProGlide utilization reached a plateau after 40 cases.

Complications during surgical interventions are related to increased hospitalization time, increased cost, and morbidity, and many previous studies in different medical disciplines focused on the correlation between LC and complications [13,14]. In this study, a significant reduction in bleeding was achieved after 40 cases. Similarly, hospitalization time was significantly decreased after 40 cases. The reduction in bleeding is believed to result in a significantly shorter hospitalization time.

Due to several factors, including not working with full rapport, unfamiliarity with the use of surgical instruments, and inexperience in the management of possible setbacks, procedure time can be longer in the first cases where a newly learned operation is performed. Sahan et al. investigated the impact of LC on stone surgery operation time, and the authors achieved a permanent decrease in operation time from the first to 15th procedures and from the 46th to 60th procedures [15]. Baytaroglu and Sevgili obtained a continuous decrease in operation time for lower extremity percutaneous thrombectomy until the 40th procedure [12]. In addition, a better understanding of anatomy and self-confidence with increasing number of cases can reduce fluoroscopy time. In this study, we achieved a significant decrease in operation time after 40 cases and in fluoroscopy time after 20 cases.

This study involves some limitations. First of all, this study presents the LC for one cardiologist about Perclose ProGlide utilization during PCI. However, every physician has a unique learning curve. For this, the outcomes of the present study should be supported by further studies. Secondly, the impact of LC on procedure cost was not a focus, which may be the subject of further research. In addition, the impact of LC on quality of life was not evaluated for Perclose ProGlide utilization during PCI.

## Conclusions

Interventional procedures are performed by experienced physicians with higher success and lower complications. For the first time, it was found that procedure time and hospitalization time significantly decreased after 40 cases, and fluoroscopy time significantly decreased after 20 cases. Moreover, after 40 procedures, the success of Perclose ProGlide utilization during PCI significantly increased and complications of the procedure significantly decreased. However, these results must be supported by further studies with larger patient volumes.

## References

[1] Hoole SP, Bambrough P (2020). Recent advances in percutaneous coronary intervention. Heart.

[2] Ellis SG, Bhatt D, Kapadia S, Lee D, Yen M, Whitlow PL (2006). Correlates and outcomes of retroperitoneal hemorrhage complicating percutaneous coronary intervention. Catheter Cardiovasc Interv.

[3] Arora N, Matheny ME, Sepke C, Resnic FS (2007). A propensity analysis of the risk of vascular complications after cardiac catheterization procedures with the use of vascular closure devices. Am Heart J.

[4] Del Prete A, Della Rocca DG, Calcagno S (2021). Perclose ProGlide™ for vascular closure. Future Cardiol.

[5] Geraci TC, Scheinerman J, Chen D, Kent A, Bizekis C, Cerfolio RJ, Zervos MD (2021). Beyond the learning curve: a review of complex cases in robotic thoracic surgery. J Thorac Dis.

[6] Bhat KG, Janardhanapillai RK, Dabas AK, Chadha DS, Swamy AJ, Chadha AS (2021). Femoral artery access site closure with perclose suture mediated device in coronary interventions. Indian Heart J.

[7] Martin JL, Pratsos A, Magargee E (2008). A randomized trial comparing compression, Perclose ProGlide and Angio-Seal VIP for arterial closure following percutaneous coronary intervention: the CAP trial. Catheter Cardiovasc Interv.

[8] Zhou Y, Shi W, Zhao D, Xiao S, Wang K, Wang J (2022). Identification of immune-associated genes in diagnosing aortic valve calcification with metabolic syndrome by integrated bioinformatics analysis and machine learning. Front Immunol.

[9] Yang S, Koo BK, Hoshino M (2021). CT angiographic and plaque predictors of functionally significant coronary disease and outcome using machine learning. JACC Cardiovasc Imaging.

[10] Saadi EK, Saadi M, Saadi R, Tagliari AP, Mastella B (2017). Totally percutaneous access using Perclose ProGlide for endovascular treatment of aortic diseases. Braz J Cardiovasc Surg.

[11] Eesa M, Burns PA, Almekhlafi MA (2014). Mechanical thrombectomy with the Solitaire stent: is there a learning curve in achieving rapid recanalization times?. J Neurointerv Surg.

[12] Baytaroglu C, Sevgili E (2022). Learning curve for percutaneous thrombectomy in treatment of acute lower extremity deep vein thrombosis. J Vasc Surg Venous Lymphat Disord.

[13] Schauer P, Ikramuddin S, Hamad G, Gourash W (2003). The learning curve for laparoscopic Roux-en-Y gastric bypass is 100 cases. Surg Endosc.

[14] Kempton LB, Ankerson E, Wiater JM (2011). A complication-based learning curve from 200 reverse shoulder arthroplasties. Clin Orthop Relat Res.

[15] Sahan M, Sarilar O, Savun M, Caglar U, Erbin A, Ozgor F (2020). Adopting for supine percutaneous nephrolithotomy: analyzing the learning curve of tertiary academic center urology team. Urology.

